# Pan-cancer analysis of DLAT reveals it as a prognostic Biomarker involved in immune infiltration of liver hepatocellular carcinoma

**DOI:** 10.7150/jca.102256

**Published:** 2025-03-21

**Authors:** Haitao Xu, Yiqun Ma, Lishi Shao, Yao Fu, Bosheng Luo, Chunjuan Xia, Xiaoli Min

**Affiliations:** 1 Department of Neurosurgery, The Second Affiliated Hospital of Kunming Medical University, Kunming, Yunnan 650101, P.R. China.; 2 Department of Radiology, 3201 Hospital, Hanzhong, Shanxi 723000, P.R. China.; 3 Department of Radiology, Yan'an Hospital Affiliated to Kunming Medical University, Kunming, Yunnan 650051, P.R. China.; 4 Department of Hepatobiliary Surgery, The Second Affiliated Hospital of Kunming Medical University, Kunming, Yunnan 650101, P.R. China.; 5 Department of Radiology, The Second Affiliated Hospital of Kunming Medical University, Kunming, Yunnan 650101, P.R. China.; 6 Department of Ultrasonography, The Second Affiliated Hospital of Kunming Medical University, Kunming, Yunnan 650101, P.R. China.; 7 Department of Cerebrovascular Diseases, The Second Affiliated Hospital of Kunming Medical University, Kunming, Yunnan 650101, P.R. China.

**Keywords:** biomarker, DLAT, liver hepatocellular carcinoma, prognosis, immune infiltration

## Abstract

**Background:** Dihydrolipoamide S-acetyltransferase (DLAT) is one of the cuproptosis-related genes (CRGs). Increasing evidence suggests that DLAT plays a critical role in various cancers. However, information about its functions and potential mechanisms in liver hepatocellular carcinoma (HCC) is still limited.

**Methods:** Bioinformatics analysis were used to evaluate the potential association of DLAT expression with N6-methyladenosine (m6A) modification, clinical features, survival prognosis, biological functions, immune infiltration, and immune checkpoint molecules (ICM) in tumor patients. In addition, the expression of DLAT in HCC tissues was verified using immunohistochemistry (IHC).

**Results:** The aberrant expression of DLAT had a significant impact on the prognosis of patients with various tumors. Importantly, DLAT expression was strongly associated with immune cell infiltration and immune checkpoint molecules (ICM) in tumors. For the first time, we found a significant positive correlation between DLAT expression and m6A regulatory factors in liver cancer, and that DLAT is associated with the PLK1 pathway, PI3K-AKT signaling pathway, Notch signaling pathway, WNT signaling pathway, and Aurora B pathway.

**Conclusions:** Our results showed a significant increase in DLAT expression in HCC. Furthermore, the prognosis of patients with high DLAT expression was poor. Importantly, DLAT was correlated with immune cell infiltration and immune checkpoint molecules in HCC patients. Together, our results indicate that DLAT represents a promising therapeutic target for HCC patients.

## Introduction

Liver cancer (LC) is commonly acknowledged as the third leading cause of cancer-related mortality worldwide, with hepatocellular carcinoma (HCC) accounting for approximately 90% of all primary liver cancers 1. The rapid progression and metastasis of HCC, coupled with delayed diagnosis in its early stages, contribute to a poor prognosis and survival rate for affected patients 2. Current therapeutic approaches for HCC include chemotherapy, gene therapy, molecular targeted therapy, and immunotherapy; however, no definitive cure is available in clinical practice, with surgical resection remaining the most effective treatment option 3. Despite advancements in treatment strategies that combine surgical resection with targeted therapies, outcomes often fall short of expectations 4. A significant proportion of individuals diagnosed with HCC present at an advanced stage where curative interventions are unfeasible, often succumbing within a year post-diagnosis 5. Therefore, investigating the pathogenesis of HCC is imperative to identify novel therapeutic targets for more effective management.

It is promising that immunotherapy has emerged as a highly effective strategy in cancer treatment 6. However, the efficacy of cancer immunotherapy depends on the ability to overcome the immunosuppressive tumor microenvironment (TME) 7. The TME comprises tumor cells, stromal components, and a substantial array of immune cells that collectively modulate tumor progression and prognosis, thus targeting the TME can enhance anti-tumor immune responses 8. The immune component of this environment is referred to as the tumor immune microenvironment (TIME), which plays a critical role in regulating tumor onset and development 9. Tumor-infiltrating immune cells are integral to the TME and can significantly influence the characteristics of TIME 10. Previous studies have demonstrated that tumor-infiltrating immune cells correlate with clinical outcomes across various solid tumors 11, 12. These findings depend on factors such as cell type, density, and the spatial distribution of infiltration 13. Furthermore, immune checkpoint blockade is an effective approach to augmenting anti-tumor T cell activity and has become one of the most promising modalities in the history of cancer therapy 14. Although checkpoint inhibitor immunotherapy for HCC is extensively utilized in major clinical trials, its clinical outcomes may be uncertain due to an immunotolerant microenvironment 15, 16. Therefore, there is an urgent need for novel therapeutic targets in HCC.

Cuproptosis, a recently identified form of cellular death induced by copper accumulation, has garnered significant interest within the oncology community, due to its potential therapeutic implications for cancer treatment 17, 18. Dihydrolipoamide S-acetyltransferase (DLAT), a key component involved in cuproptosis, and an E2 subunit of the multienzyme pyruvate dehydrogenase complex, has been implicated in various oncogenic processes based on several investigations 19-21. Nevertheless, its precise role in HCC remains largely unexplored.

In this study, we employed bioinformatics analyses to assess DLAT expression levels within tumors and their impact on patient prognosis. To investigate the underlying causes of abnormal DLAT expression in tumors, we examined correlations between DLAT levels, DNA methylation, and m6A-related genes. We utilized gene ontology (GO) 22, Kyoto Encyclopedia of Genes and Genomes (KEGG) 23, and Gene Set Enrichment Analysis (GSEA) 24 to predict potential roles for DLAT in HCC. We evaluated associations between DLAT expression and immune infiltration using "CIBERSORT" 25 and "ssGSEA" 26 algorithms. Additionally, immunohistochemistry (IHC) was conducted to validate DLAT expression in HCC tissues. Based on these findings, we propose that DLAT represents a promising novel therapeutic target for HCC.

## Materials and methods

### Data processing and differential expression analysis

We obtained the standardized pan-cancer dataset from the University of California, Santa Cruz Genomic Browser (UCSC) database (https://xenabrowser.net/) 27: TCGA TARGET GTEx data (PANCAN, N=19131, G=60499). Subsequently, we extracted gene expression data for ENSG00000150768 (DLAT) from all samples in the TCGA dataset. The TCGA-LIHC (liver hepatocellular carcinoma) cohort data, including clinical information for 374 HCC patients and 50 non-tumor patients, was downloaded from The Cancer Genome Altas (TCGA) website (https://portal.gdc.cancer.gov) 28. We used the Tumor Immune Estimation Resource (TIMER) database (https://cistrome.shinyapps.io/timer/) 29 to analyze DLAT expression across 33 distinct cancer types. The GSE121248 and GSE54236 cohort data from the Gene Expression Omnibus (GEO) database (https://www.ncbi.nlm.nih.gov/geo/) 30 were used to analyze DLAT expression in HCC. We analyzed the DNA methylation levels of DLAT in pan-cancer using the University of Alabama at Birmingham Cancer Data Analysis Portal (UALCAN) database (https://ualcan.path.uab.edu) 31: the analysis included data from 793 patients with invasive breast carcinoma (BRCA), 502 with prostate adenocarcinoma (PRAD), 528 with head and neck squamous cell carcinoma (HNSC), 473 with lung adenocarcinoma (LUAD), 313 with colon adenocarcinoma (COAD), and 98 with rectal adenocarcinoma (READ). The Wilcoxon rank sum test was used to compare two sets of data, with significance defined as P < 0.05.

### Correlation between DLAT expression and clinicopathological features in pan-cancer

The correlations between DLAT expression and clinicopathological features were evaluated using the TCGA database, which included pathologic stage, World Health Organization (WHO) grade, isocitrate dehydrogenase (IDH) status, pathologic N stage, pathologic M stage, and T stage. A total of 374 patients with HCC, 701 patients with glioma (GBMLGG), 647 patients with colon adenocarcinoma/rectum adenocarcinoma esophageal carcinoma (COADREAD), 1,113 patients with BRCA, 541 patients with kidney renal clear cell carcinoma (KIRC), and 179 patients with pancreatic adenocarcinoma (PAAD) participated in the analysis. The inclusion criteria for patients with HCC were as follows: the primary lesion must be HCC, and patients with incomplete follow-up information were excluded. The inclusion criteria for patients with GBMLGG, COADREAD, BRCA, KIRC, and PAAD are consistent with those described above for HCC patients.

### Overall survival (OS) outcome assessment

We acquired DLAT expression data and clinically relevant information for tumors from the TCGA databases. We analyzed the effect of DLAT on the prognosis of tumor patients, including 33 cancer types in the pan-cancer prognosis analysis. The survival package (version 3.3.1) was used to evaluate the proportional hazards assumption and conduct survival regression analysis, with results visualized using the survminer and ggplot2 packages.

### Immune cell infiltration assessment

To comprehensively investigate whether DLAT expression is associated with immune cell infiltration in HCC, we analyzed immune cell infiltration data from the TCGA database. The relationship between DLAT expression and immune cell infiltration was assessed using the "CIBERSORT" and "ssGSEA" algorithms. We downloaded and organized the STAR process RNAseq data and clinical data from the TCGA database for the TCGA-LIHC (HCC) project, extracting the data in TPM format. Correlation analysis was performed between principal variables and immune infiltration matrix data in the data, and the analysis results were visualized using ggplot2 package [3.3.6].

### Enrichment analysis

GO and KEGG analyses were based on differentially expressed genes (with adj. p-value < 0.01 and |logFC| ≥ 2; total = 94) between high and low DLAT expression in HCC tissues, using the "clusterProfiler" R package to explore the potential biological functions and signaling pathways affected by DLAT. Subsequently, GSEA was conducted to identify possible signaling pathways involving DLAT in HCC. The gene sets (c2.cp.kegg.v2022.1.Hs.symbols.gmt), which served as reference gene sets, were downloaded from the Molecular Signatures Database (MSigDB) (https://www.gsea-msigdb.org) 32. Significance was considered when the p-value was less than 0.05.

### IHC

We collected thirty-six cases of HCC along with their associated paracancerous tissues from the Second Affiliated Hospital of Kunming Medical University, following radical surgical resection. All patients were diagnosed according to the diagnostic and treatment guidelines established by the European Association for the Study of the Liver (EASL). This study was approved by the Ethics Committee of the Second Affiliated Hospital of Kunming Medical University. All patients provided written informed consent. The inclusion criteria for HCC patients were as follows: (1) the primary lesion must be HCC, and (2) patients with other diseases were excluded. To assess the protein expression level of DLAT gene in six pairs of HCC and corresponding paracancerous tissues, we conducted an immunohistochemical analysis. After drying at 60°C for 3 hours, paraffin-embedded tissue sections were dewaxed in xylene and subsequently hydrated with a gradient of alcohol. The tissue sections were then subjected to high-pressure heating in citrate antigen retrieval solution (MVS-0100, Fuzhou Maixin Biotech, ltd, China) for antigen retrieval. Following H_2_O_2_ treatment and blocking with 10% normal goat serum, the slides were incubated overnight with the primary antibody, anti-DLAT (ABclonal Technology, A8814). The corresponding secondary antibody was anti-Rabbit IgG (K5007, Agilent Technologies, United States of America). The staining was performed using a diaminobenzidine (DAB) kit (K5007, Agilent Technologies, United States of America), and hematoxylin was applied for counterstaining. The slides were examined under a laboratory microscope (Nikon, Japan). The immunostaining results was evaluated by multiplying stain intensity by the percentage of positive cells [staining index (SI)]. Stain intensity was divided into four categories as follows: no staining (score 0), weak staining (score 1), moderate staining (score 2), or strong staining (score 3). The percentage of positive cells was divided into four categories as follows: <25 % (score 1), 25-50 % (score 2), 50-75 % (score 3), or more than 75 % (score 4). The expression levels of DLAT were determined by the SI, which scores as 0, 1, 2, 3, 4, 6, 8, 9, and 12. A cutoff value was identified as follows: the median SI score of 4 or greater was used to define tumors as high DLAT expression and the SI score of 3 or less as low DLAT expression. DLAT slides were classified as positive (SI = 4 and 4+) and negative (SI = 0-3).

### Statistical analysis

Statistical analyses of the datasets from the TCGA database were performed using R software (version 4.2.1). The log-rank test was used for survival analysis, while the relationships between genes were evaluated using p-values and Spearman correlation coefficients. The Wilcoxon rank-sum test was employed for continuous variables, and the chi-square test was used for categorical variables to assess the correlation between DLAT and pathological features. A statistically significant result was defined as a p-value of less than 0.05.

## Results

### Expression analysis of DLAT in pan-cancer

**Figure 1:**
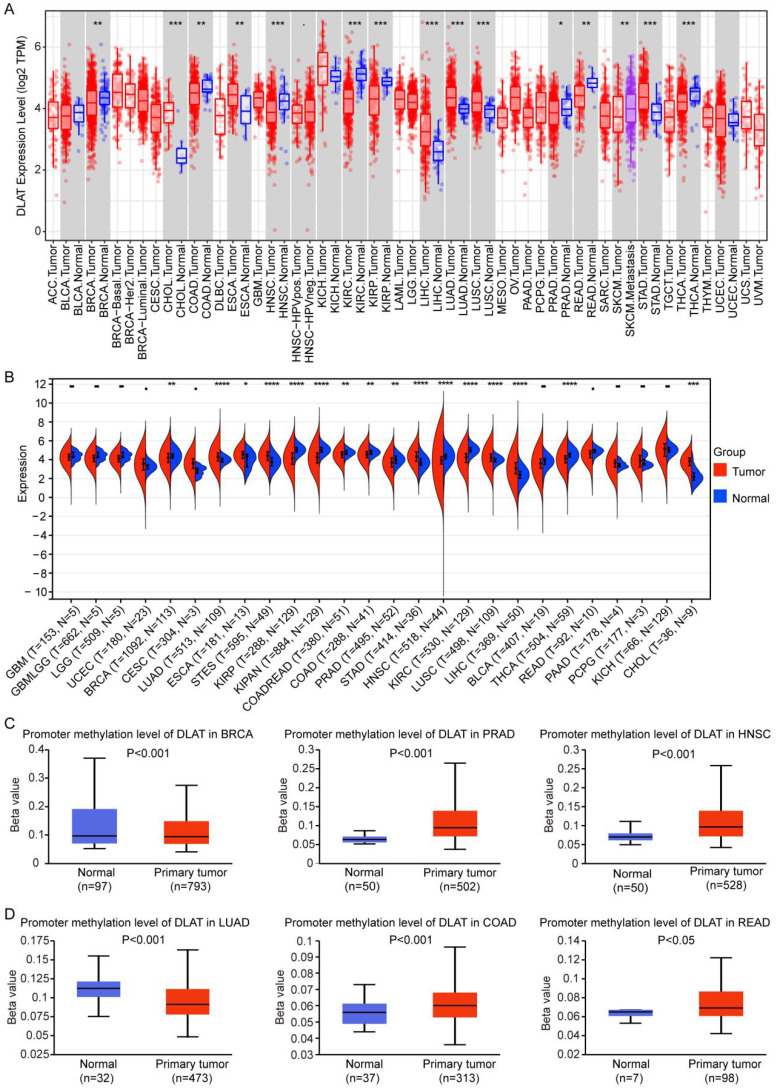
DLAT was abnormally expressed in pan-cancer. (A-B) The expression of DLAT in pan-cancer was analyzed by using TIMER (A) and TCGA (B) databases. (C-D) DNA methylation levels of DLAT in pan-cancer were analyzed by UALCAN database. *, P < 0.05, **, P < 0.01, ***, P < 0.001, ****, P < 0.001.

Through the joint analysis of TIMER and TCGA databases, we found that DLAT was highly expressed in cholangiocarcinoma (CHOL), esophageal cancer (ESCA), liver hepatocellular carcinoma (LIHC), LUAD, lung squamous cell carcinoma (LUSC), and stomach adenocarcinoma (STAD), and was lowly expressed in BRCA, COAD, HNSC, KIRC, kidney renal papillary cell carcinoma (KIRP), READ, PRAD, and thyroid cancer (THCA) (Figure 1A-B). DNA methylation regulates gene expression levels 33. To explore the reasons for the abnormal expression of DLAT in various tumors, we analyzed the DNA methylation levels of DLAT in tumors and found that DLAT exhibited high methylation levels in PRAD, COAD, READ, and HNSC, and low methylation levels in BRCA and LUAD (Figure 1C-D). The expression of DLAT was not negatively correlated with DNA methylation levels in breast cancer. Reports indicate that in breast cancer, DNA methylation and histone modification are mutually exclusive processes, which may lead to diminished expression levels of certain genes in regions characterized by low methylation 34.

**Figure 2:**
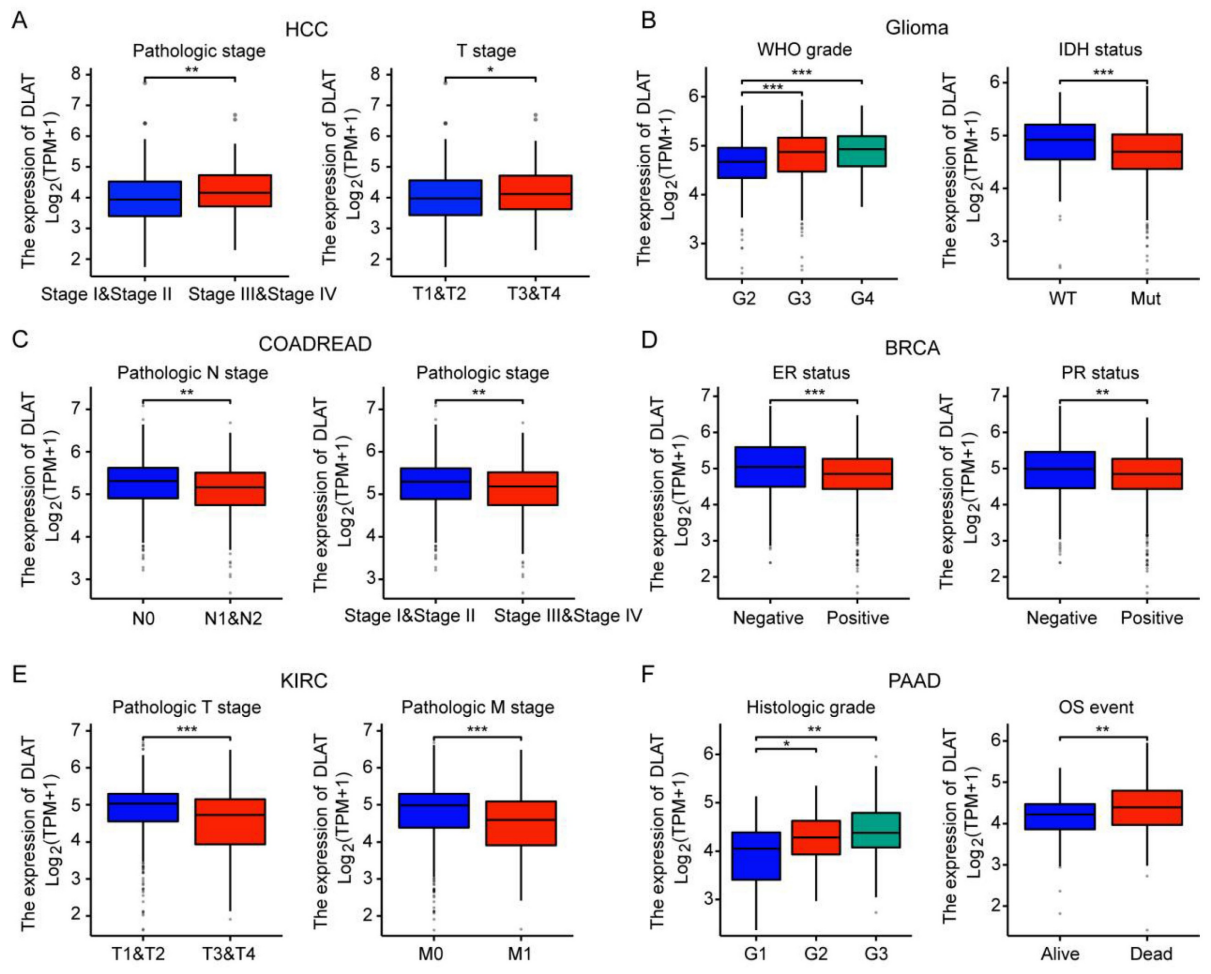
Correlation between the DLAT expression and clinicopathologic features in pan-cancer. DLAT was significantly correlated with pathologic stage and T stage in HCC (A), WHO grade and IDH status in GBMLGG (B), pathologic N stage pathologic stage in COADREAD (C), PR status and ER status in BRCA (D), pathologic T stage and pathologic M stage in KIRC (E) and histologic grade and OS event in PAAD (F). *, P < 0.05, **, P < 0.01, ***, P < 0.001.

Subsequently, we analyzed the association of DLAT with the clinicopathological features of major tumors. In HCC, DLAT expression levels were significantly elevated in stages III and IV as well as T3 and T4 stages compared to stages I and II, and T1 and T2 stages. In GBMLGG, DLAT expression positively correlated with tumor grade; notably, IDH wild-type gliomas exhibited higher DLAT levels than their IDH mutant counterparts. Conversely, in COADREAD, DLAT expression was greater in stages I and II and N0 stage compared to stages III and IV or N1 and N2 stages. Furthermore, BRCA demonstrated lower DLAT expression in ER-positive and PR-positive subtypes relative to ER-negative and PR-negative variants. In KIRC, elevated DLAT levels were observed at T1 and T2 stages alongside M0 stage when compared to T3 and T4 or M1 stages. Lastly, PAAD showed that increased DLAT expression was associated with higher histologic grade; in addition, survivors of PAAD displayed lower expression levels of DLAT than non-survivors (Figure 2A-F). The results imply that DLAT may serve as a significant biomarker in cancer diagnosis.

### Prognostic analysis of DLAT in pan-cancer

**Figure 3:**
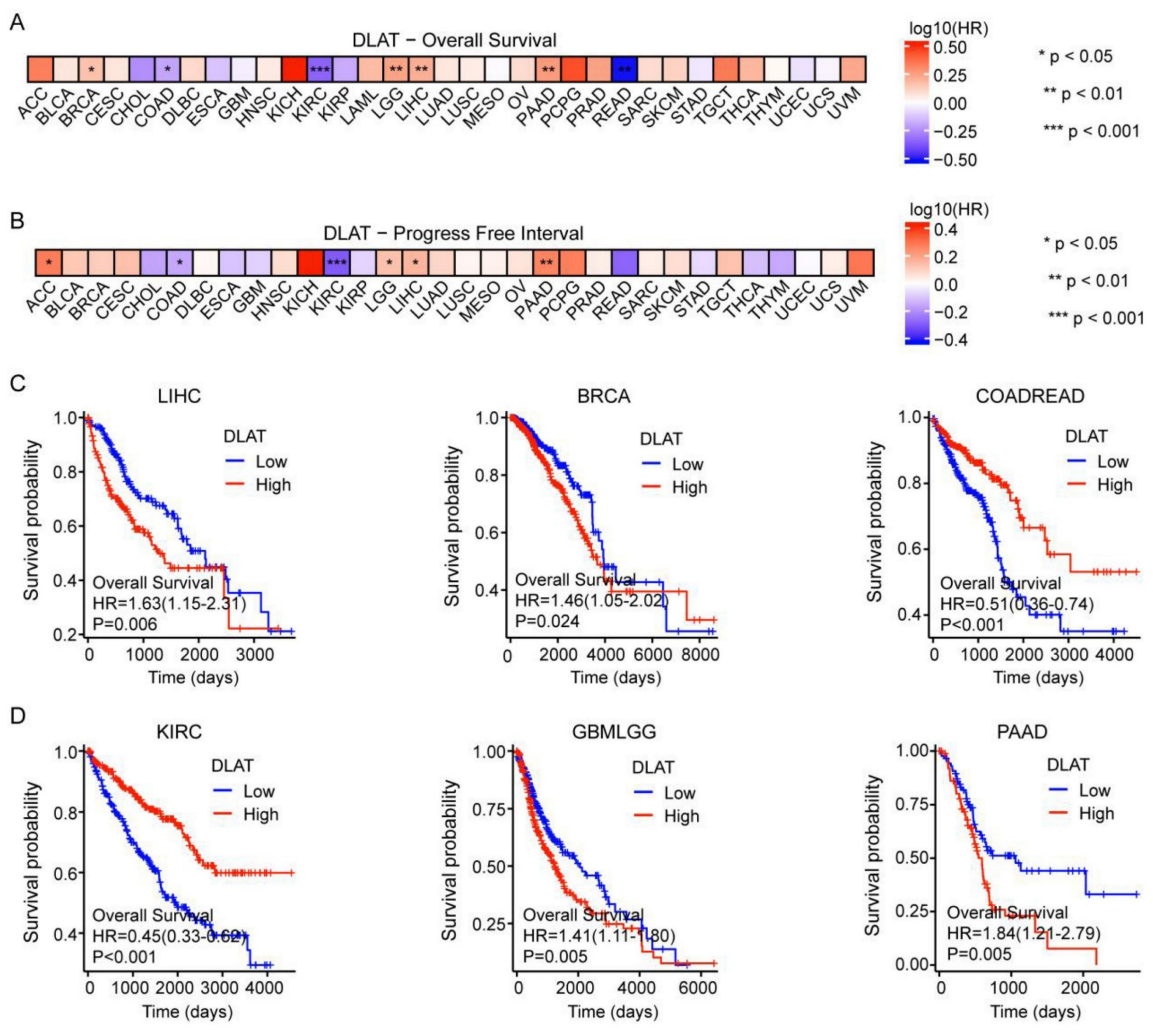
The effect of DLAT on the prognosis of tumor patients. (A-B) The effect of DLAT expression on OS and PFI in pancarcinoma was analyzed using Cox regression analysis. (C-D) The Kaplan-Meier (KM) analysis revealed the impact of DLAT expression on OS in patients with LIHC, BRCA, COADREAD, KIRC, GBMLGG, and PAAD.

We next classified patients into elevated and reduced DLAT cohorts based on the median DLAT expression levels within samples from the TCGA database. In terms of overall survival (OS), DLAT served as a risk factor in BRCA, LGG, LIHC, and PAAD, according to Cox regression analyses. In contrast, it acted as a protective factor for COAD, KIRC, and READ. Subsequently, Cox regression analysis of progression-free interval (PFI) indicated that DLAT was a risk factor for four distinct cancers: adrenocortical carcinoma (ACC), brain lower grade glioma (LGG), LIHC, and PAAD. Conversely, it served as a protective factor for COAD and KIRC (Figure 3A-B and Figure S1). The Kaplan-Meier (KM) analysis on the OS illustrated that high levels of DLAT expression were related to dismal prognoses in patients suffering from LIHC, BRCA, GBMLGG, and PAAD, but a favorable prognosis was found in COADREAD and KIRC (Figure 3C-D). As shown in Table 1, patients with complete clinical data were included in the subsequent Cox regression analysis. Before Cox regression analysis, we would remove samples with missing values in the outcome or time columns, as samples with missing values could not be included in the analysis. Cox univariate regression analysis indicated that DLAT, pathological stage, pathological T stage, and pathological M stage were associated with overall survival in HCC patients. Furthermore, multivariate Cox analysis revealed that DLAT was an independent prognostic factor for overall survival in HCC patients.

### The significance of DLAT in TIME

**Figure 4:**
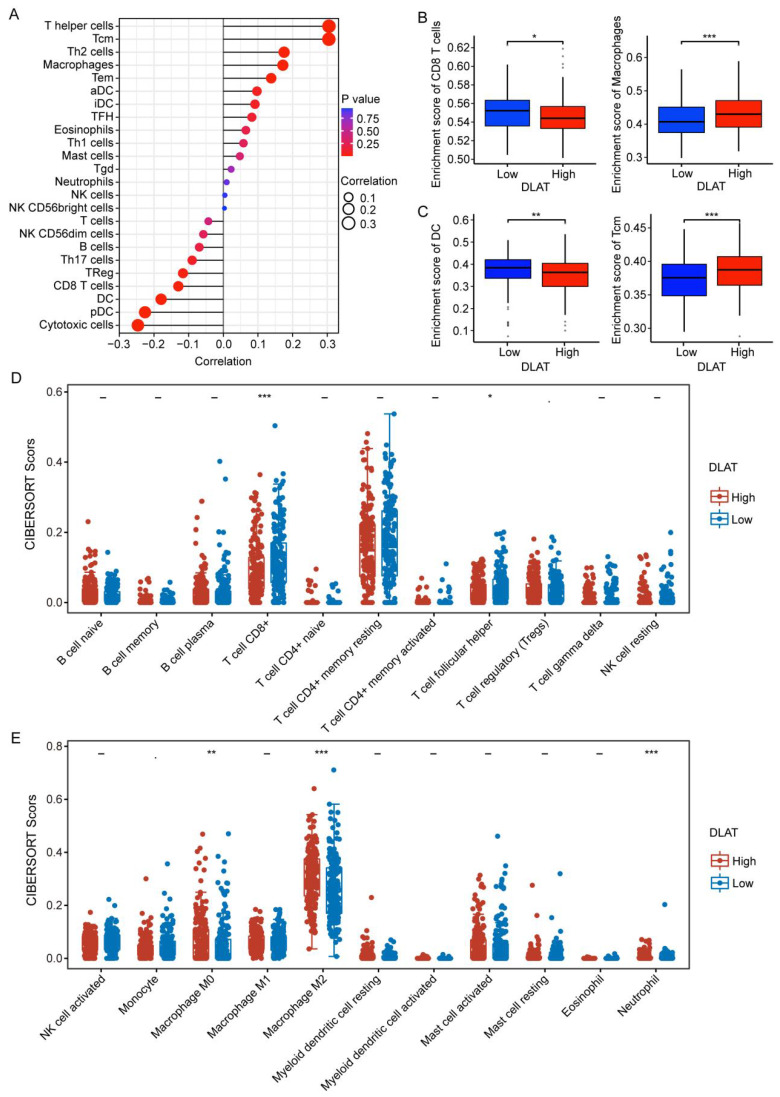
Association between DLAT and immune infiltration in patients with HCC. (A-C) Association between DLAT and immune infiltration in patients with HCC using "ssGSEA" algorithm. (D-E) Association between DLAT and immune infiltration in patients with HCC using the "CIBERSORT" algorithm. * P < 0.05, ** P < 0.01, *** P < 0.001.

An increasing number of reports underscore the significance of the tumor immune microenvironment (TIME), which induces immunosuppression and accelerates tumor progression 35. Herein, we investigated the relationship between DLAT and immune infiltration in HCC using multiple algorithms. Using the "ssGSEA" algorithm, we found that DLAT was significantly positively associated with macrophages, activated dendritic cells (aDC), mast cells, eosinophils, T helper cells, central memory T cells (Tcm), T helper 2 cells (Th2), effector memory T cells (Tem), T helper 1 cells (Th1), and immature dendritic cells (iDC), while negatively associated with CD8+ T cells, regulatory T cells (Treg), T helper 17 cells (Th17), B cells, T cells, dendritic cells (DC), plasmacytoid dendritic cells (pDC), cytotoxic cells, and NK CD56dim cells (Figure 4A-C). Next, we verified these findings using the "CIBERSORT" algorithm (Figure 4D-E). These findings suggest that DLAT plays a critical role in the immune invasion of HCC, paving the way for the identification of novel and effective bioindicators for HCC immunotherapy.

**Figure 5:**
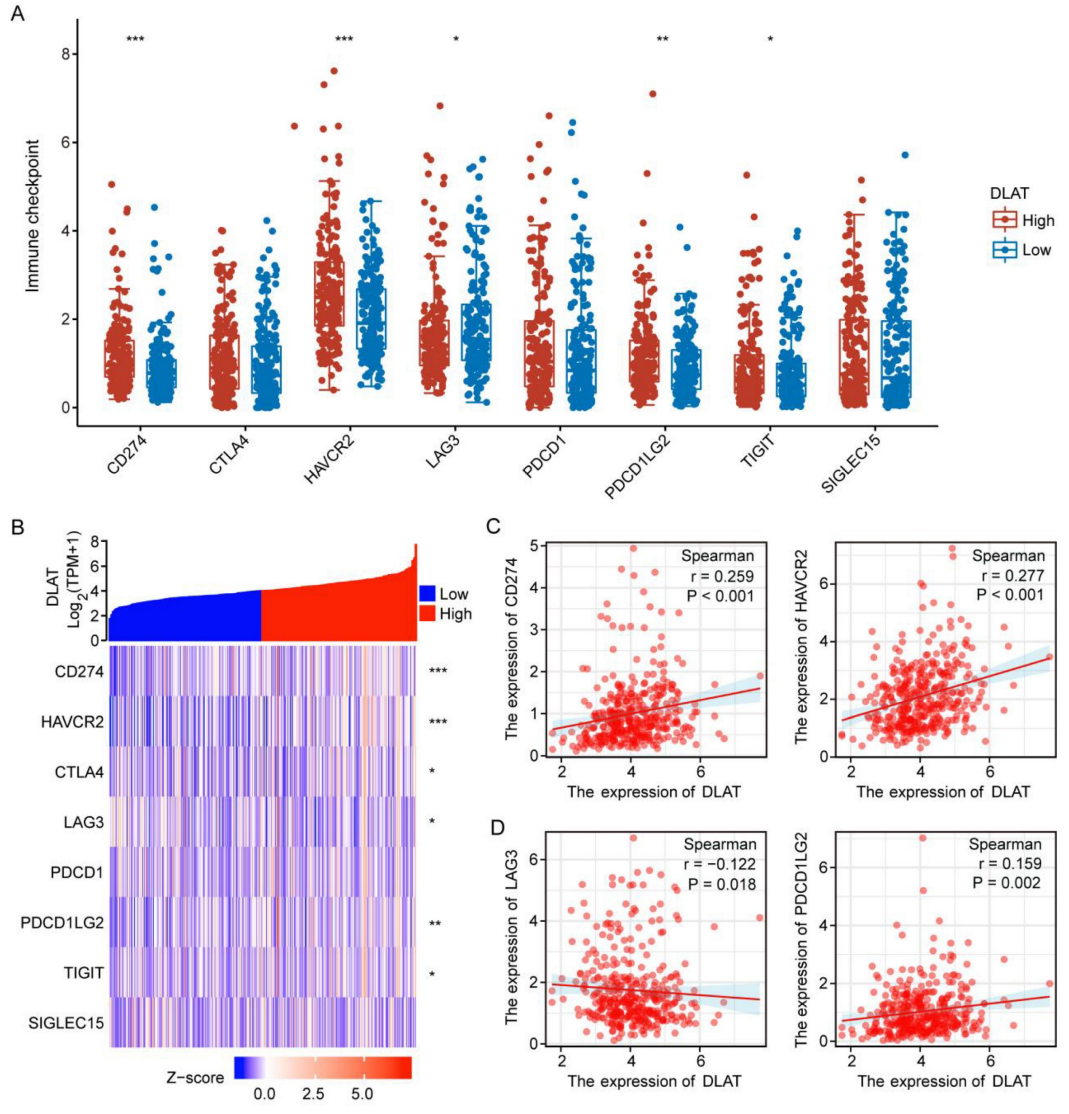
The association between DLAT expression and immune checkpoint molecules. (A-B) Correlation between DLAT expression and immune checkpoint genes in patients with HCC using TCGA databases. (C-D) Analyzing the correlation between DLAT expression and immune checkpoint genes in patients with HCC using Spearman's correlation. * P < 0.05, ** P < 0.01, *** P < 0.001.

Immune checkpoints are molecules that inhibit immune responses to prevent autoimmunity 36. Herein, we investigated the correlation between DLAT expression and immune checkpoint molecules (ICM) in HCC. The analysis revealed that DLAT was positively correlated with several immune checkpoint molecules, including CD274, HAVCR2, and PDCD1LG2, while showing a negative correlation with LAG3 (Figure 5A-D). Collectively, this evidence confirms the significant role of DLAT in HCC immunotherapy.

### GSEA of DLAT in patients with HCC

**Figure 6:**
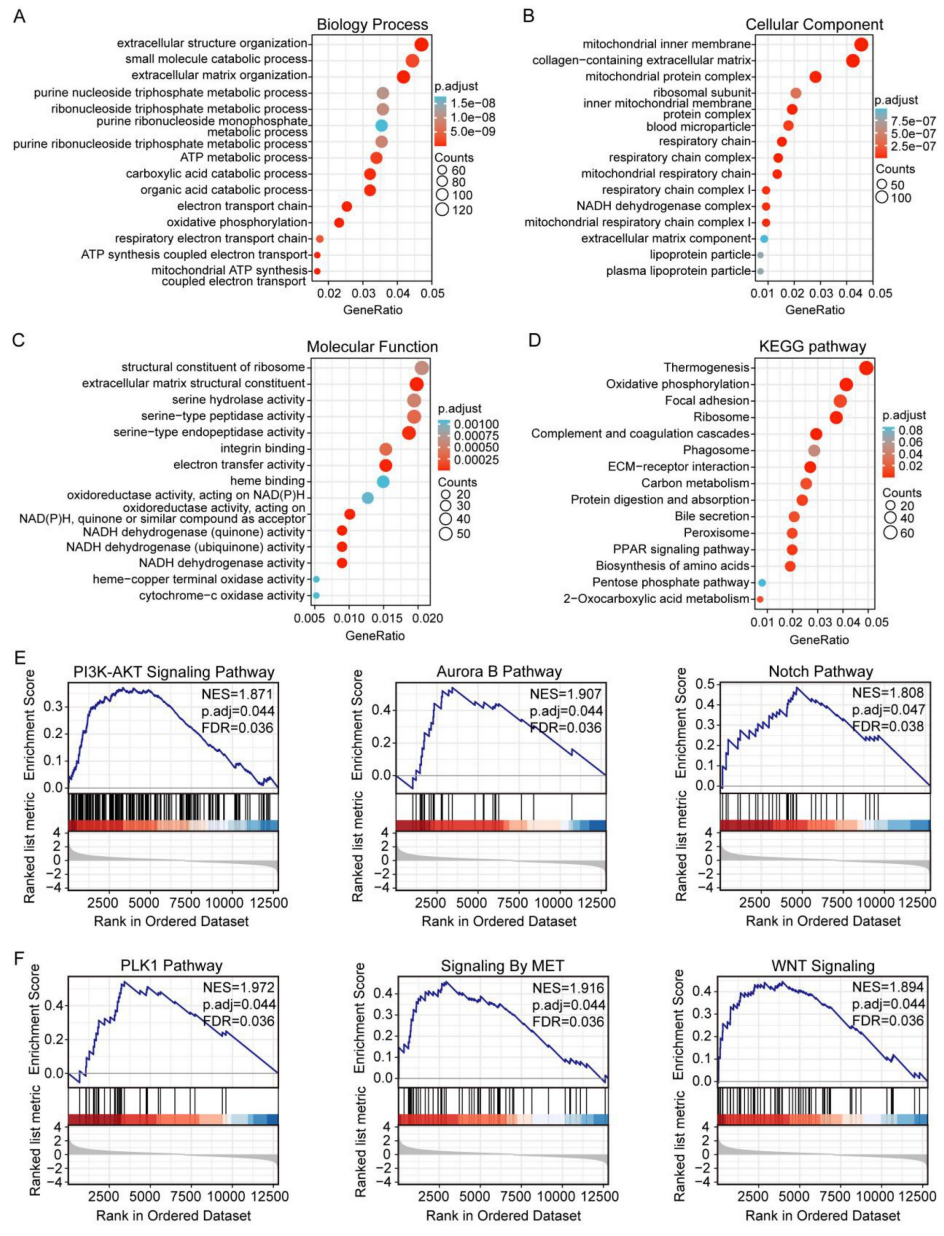
GO and KEGG enrichment analysis of DLAT in HCC. (A-C) The GO enrichment analysis was performed on DLAT using TCGA. (D) The KEGG pathway analysis of DLAT using the TCGA database. (E-F) GSEA enrichment analysis showed that the PI3K-Akt signaling pathway, aurora B pathway, Notch pathway, PLK1 pathway, MET signaling pathway and Wnt signaling pathway were enriched by DLAT. * P < 0.05, ** P < 0.01, *** P < 0.001.

Our studies have shown that increased DLAT expression is associated with poor prognosis in HCC patients. To further explore the impact of DLAT expression in HCC, we conducted GO and KEGG enrichment analyses to elucidate the functional role of DLAT. The biological process (GO-BP) terms enriched by DLAT included extracellular structure organization, small molecule catabolism, extracellular matrix (ECM) organization, purine nucleoside triphosphate metabolism, ribonucleoside triphosphate metabolism, ATP metabolism, carboxylic acid catabolism, organic acid catabolism, as well as electron transport chain and oxidative phosphorylation processes (Figure 6A). The cellular component (GO-CC) terms enriched by DLAT included mitochondrial inner membrane, collagen-possessing ECM, mitochondrial protein complex, ribosomal subunit, inner mitochondrial membrane protein complex, blood microparticle, respiratory chain, respiratory chain complex, mitochondrial respiratory chain, respiratory chain complex I, and NADH dehydrogenase complex (Figure 6B). The molecular function (GO-MF) terms enriched by DLAT included structural constituent of ribosome, ECM structural constituent, as well as activities such as serine hydrolase, serine-type peptidase, serine-type endopeptidase, integrin binding, electron transfer, heme binding, oxidoreductase activities, and targeting NAD(P)H and quinones or similar compounds that serve as acceptors (Figure 6C).

The KEGG analysis revealed that DLAT is enriched in pathways including thermogenesis, oxidative phosphorylation, focal adhesion, ribosome biogenesis, complement and coagulation cascades, phagosome, ECM-receptor interaction, carbon metabolism, protein digestion and absorption, bile secretion, peroxisome, PPAR signaling pathway, and the pentose phosphate pathway (Figure 6D).

Finally, GSEA was utilized to examine DLAT-associated networks. We revealed that DLAT contributed to the PI3K-AKT, Aurora B, Notch, PLK1, MET, and Wnt signaling pathways (Figure 6E-F). Notably, most of these pathways are linked to the immune response. The PI3K-AKT signaling pathway may impair immune cell effector functions and alter the TME 37. Mutations in the Notch signaling pathway have been suggested as crucial biomarkers for immune checkpoint blockade therapy in various cancers 38. PLK1 promotes the polarization of M2 macrophages by interacting with PTEN 39. The Wnt pathway strongly regulates immune cell infiltration in the TME 40. MET signaling involves the immune response and mainly regulates the function of dendritic cells 41.

### Associations between N6-methyladenosine RNA methylation and DLAT expression in HCC

**Figure 7:**
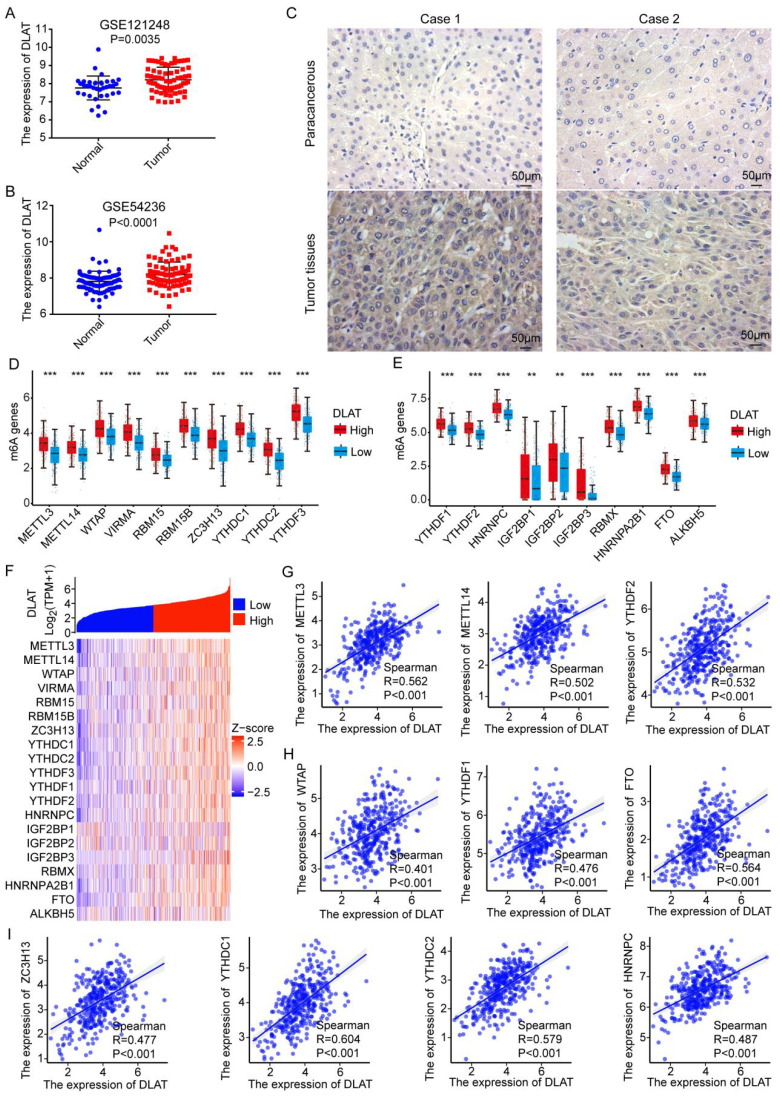
Association Between N6-methyladenosine RNA Methylation and DLAT Expression in HCC. (A-B) The high expression of DLAT in HCC was demonstrated by GEO datasets. (C) The expression level of DLAT in HCC tissues was verified by immunohistochemistry. Scale bar =50 um. (D-F) Association between DLAT expression and m6A-related genes in patients with HCC using TCGA database. (G-I) Analyzing the association between DLAT expression and m6A-related genes in patients with HCC using Spearman' s correlation. * P < 0.05, ** P < 0.01, *** P < 0.001.

To validate our previous results, we analyzed DLAT expression in hepatocellular carcinoma (HCC) using the GEO database and found that DLAT is highly expressed in HCC (Figure 7A-B). Additionally, we found that the expression of DLAT is significantly associated with the clinical pathological features of liver cancer patients, including pathologic T stage and pathological stage (Table 2). Subsequence, we used immunohistochemistry to confirm that the protein expression level of DLAT was higher in HCC tissue compared to adjacent non-cancerous tissue (Figure 7C). RNA methylation plays a crucial role in regulating gene expression 42. To elucidate the mechanism behind the abnormally high expression of DLAT in HCC, we investigated the association between RNA methylation and DLAT levels. We observed a significant positive correlation between DLAT expression and m6A-associated genes, namely METTL3 (R=0.562, P<0.001), METTL14 (R=0.502, P<0.001), YTHDF2 (R=0.532, P<0.001), WTAP (R=0.401, P<0.001), YTHDF1 (R=0.476, P<0.001), FTO (R=0.564, P<0.001), ZC3H13 (R=0.477, P<0.001), YTHDC1 (R=0.604, P<0.001), YTHDC2 (R=0.579, P<0.001), HNRNPC (R=0.487, P<0.001) (Figure 7G-I). Clearly, this evidence demonstrates that DLAT strongly modulates HCC development by interacting with RNA methylation-related genes.

## Discussion

Despite significant advancements in HCC research, the critical factors contributing to HCC development remain poorly understood. Most patients are diagnosed with advanced-stage HCC at diagnosis, resulting in a 5-year overall survival rate of less than 10%. Recurrence and metastasis of HCC are pivotal factors contributing to the suboptimal therapeutic outcomes observed in this malignancy. Chemotherapy and molecular targeted therapies are the primary interventions for advanced HCC. However, tumor drug resistance and treatment-related adverse reactions significantly hinder the efficacy of these targeted therapies. Therefore, identifying novel therapeutic targets for HCC is essential.

As public tumor-related databases continue to evolve, new immunotherapeutic targets can be identified through comprehensive pan-cancer analyses of individual genes. These analyses evaluate correlations with clinicopathological characteristics, clinical outcomes, immune infiltration patterns, and relevant signaling pathways. Consequently, the development of new biomarkers for cancer diagnosis and prognosis is imperative. Our findings indicate that DLAT expression is markedly elevated in several cancers, such as HCC, CHOL, ESCA, COAD, LUAD, and LUSC, and correlates with various clinicopathological features. Prognostic analysis revealed that increased DLAT levels are associated with poor outcomes in HCC, GBMLGG, and PAAD. Furthermore, multivariate Cox regression analysis identified DLAT as an independent prognostic factor for HCC. Thus, we selected this context for subsequent functional enrichment analysis and immunohistochemical validation to confirm the expression of DLAT.

We employed bioinformatics approaches to elucidate the significance of DLAT in tumors by analyzing its expression profile along with prognostic indicators such as immune infiltration patterns and checkpoint interactions. Our results demonstrated that aberrant expression levels of DLAT strongly correlated with clinicopathological features. Additionally, immunohistochemistry analysis confirmed that DLAT protein levels were significantly higher in HCC tissues than in adjacent non-cancerous tissues. The GO-BP terms enriched by DLAT included extracellular structure organization and metabolic processes related to small molecules, such as ATP metabolism, purine nucleoside triphosphate metabolism, and oxidative phosphorylation pathways. The GO-CC terms enriched by DLAT included mitochondrial inner membranes and components involved in collagen-rich extracellular matrices. The GO-MF terms highlighted roles such as ribosomal structural constituents and enzymatic activities, including serine hydrolase activity targeting NAD(P)H compounds or similar acceptors. KEGG pathway analysis demonstrated enrichment in thermogenesis, oxidative phosphorylation, focal adhesion, complement coagulation cascades, phagosome, ECM-receptor interactions, carbon metabolism, and the PPAR signaling pathway. Ultimately, we analyzed the signaling pathways involved in DLAT using GSEA. The results showed that DLAT was involved in the PI3K-AKT signaling pathway, the aurora B pathway, the Notch pathway, the PLK1 pathway, the MET signaling pathway, and the Wnt signaling pathways. These signaling pathways involved were significantly associated with the immune response 37-41, providing a new strategy for studying the immune role of DLAT in liver cancer.

Research has shown that m6A RNA methylation affects gene expression and is involved in a variety of human cancers, including HCC 43. In HCC, METTL3 promotes tumor growth by regulating oncogenic and tumor-suppressing genes, including SNAIL, YAP1, LINC00958, and SOCS2. In contrast, METTL14 inhibits HCC growth by upregulating USP48, HNF3γ, and miR126, while silencing SLC7A11. In this study, we found that DLAT expression was significantly positively correlated with m6A methylation regulators, including METTL3, METTL14, YTHDF2, WTAP, YTHDF1, FTO and ZC3H13, etc 44. Therefore, the crosstalk between m6A RNA modification and DLAT expression in liver cancer needs to be further characterized in future studies, which will be critical to evaluate the potential of using m6A and its regulators for early cancer diagnosis.

Immunotherapy is revolutionizing cancer treatment 45, particularly in the widespread management of HCC patients 46. However, a lack of biomarkers to predict the efficacy and outcome of HCC immunotherapy makes the discovery of effective immunotherapy markers an urgent priority 47. Using the "ssGSEA" and "CIBERSORT" algorithms, we analyzed the relationship between DLAT expression and immune cell infiltration. Our findings revealed a significant positive correlation between DLAT and several immune cell types, including macrophages, activated dendritic cells (aDC), mast cells, eosinophils, T helper cells, central memory T cells (Tcm), T helper 2 cells (Th2), effective memory T cells (Tem), T helper 1 cells (Th1), and immature dendritic cells (iDC). Conversely, DLAT showed a negative association with CD8+ T cells, regulatory T cells (Treg), T helper 17 cells (Th17), B cells, dendritic cells (DC), plasmacytoid dendritic cells (pDC), cytotoxic cells, and NK CD56dim cells. With advancements in research on tumor immune escape mechanisms, immunotherapy using immune checkpoint inhibitors (ICI) has been widely implemented across various tumor types, which expands the treatment landscape for advanced HCC 48. Our analysis revealed a positive correlation between DLAT and several immune checkpoint molecules, including CD274, HAVCR2, and PDCD1LG2, as well as a negative correlation with LAG3. These findings suggest that DLAT may serve as a promising immunotherapy marker for HCC.

## Conclusions

In conclusion, the findings of this study demonstrate that DLAT is highly expressed in HCC. Additionally, patients with elevated DLAT levels exhibit a poor prognosis. Importantly, DLAT is associated with immune cell infiltration and immune checkpoint molecules in HCC patients. Together, our results suggest that DLAT represents a promising therapeutic target for HCC patients.

## Supplementary Material

Supplementary figure and table.

## Figures and Tables

**Table 1:** Univariate and Multivariate analyses of the association between clinicopathologic features and overall survival.

Characteristics	Total(N)	Univariate analysis		Multivariate analysis	
		Hazard ratio (95% CI)	P value	Hazard ratio (95% CI)	P value
Pathologic T stage	370				
T1&T2	277	Reference		Reference	
T3&T4	93	2.598 (1.826 - 3.697)	< 0.001	1.916 (0.260 - 14.140)	0.524
Pathologic M stage	272				
M0	268	Reference		Reference	
M1	4	4.077 (1.281 - 12.973)	0.017	2.071 (0.635 - 6.754)	0.227
Pathologic stage	349				
Stage I&Stage II	259	Reference		Reference	
Stage III&Stage IV	90	2.504 (1.727 - 3.631)	< 0.001	1.419 (0.193 - 10.445)	0.731
Age	373				
<= 60	177	Reference			
> 60	196	1.205 (0.850 - 1.708)	0.295		
DLAT	373				
Low	186	Reference		Reference	
High	187	1.627 (1.148 - 2.307)	0.006	1.610 (1.042 - 2.487)	0.032

The reasons for missing cases: If a categorical variable has more than 10 categories, it cannot be identified as a categorical or ordinal variable; If a variable is encoded with groups like 0, 1, 2, and the number of categories is less than 5, it will be recognized as a categorical variable; if greater than 5, it will be identified as a numerical variable; If the data contains infinite values, those infinite values will be treated as missing values; Additional note: Before conducting univariate analysis, samples with missing values in the outcome and time columns will be removed (samples with missing time or outcome cannot be included in the analysis); Missing value handling strategy: Handle variable missingness after univariate analysis and before multivariate analysis.

**Table 2:** The correlation between DLAT expression and clinic parameters

Characteristics	Low expression of DLAT	High expression of DLAT	pvalue
n	120	118	
Pathologic T stage, n (%)			0.007702627
T1&T2	95 (39.9%)	75 (31.5%)	
T3&T4	25 (10.5%)	43 (18.1%)	
Pathologic M stage, n (%)			1
M0	118 (49.6%)	116 (48.7%)	
M1	2 (0.8%)	2 (0.8%)	
Pathologic N stage, n (%)			0.602211043
N0	119 (50%)	115 (48.3%)	
N1	1 (0.4%)	3 (1.3%)	
Pathologic stage, n (%)			0.00865185
Stage I&Stage II	93 (39.1%)	73 (30.7%)	
Stage III&Stage IV	27 (11.3%)	45 (18.9%)	
Age, n (%)			0.611384773
<= 60	68 (28.6%)	63 (26.5%)	
> 60	52 (21.8%)	55 (23.1%)	

From the TCGA database (https://portal.gdc.cancer.gov) to download and organize TCGA - LIHC (HCC) project STAR process RNAseq data and extract the TPM format of the data and clinical data. We selected patients with hepatocellular carcinoma only and excluded patients with other diseases. The reasons for missing cases: If a categorical variable has more than 10 categories, it cannot be identified as a categorical or ordinal variable; If a variable is encoded with groups like 0, 1, 2, and the number of categories is less than 5, it will be recognized as a categorical variable; if greater than 5, it will be identified as a numerical variable. If the data contains infinite values, those infinite values will be treated as missing. Missing value handling strategy: Remove samples with any missing variables.
