# Supplementary material for: Pan-cancer analysis of DLAT reveals it as a prognostic Biomarker involved in immune infiltration of liver hepatocellular carcinoma
Source: J Cancer. 2025 Mar 21;16(7):2167–80. doi: 10.7150/jca.102256 (PMC12036108; doi:10.7150/jca.102256)
Supplement: Supplementary file 1 — Supplementary figure and table. [file jcav16p2167s1.pdf]

Supplementary Material

A

DLAT − Overall Survival

Group	Total(N)	HR(95% CI)	P value
ACC	79	2.172 (1.000 ... 4.716)	0.0500
BLCA	411	1.163 (0.868 ... 1.557)	0.3115
BRCA	1086	1.456 (1.050 ... 2.018)	0.0242
CESC	306	1.171 (0.736 ... 1.862)	0.5057
CHOL	35	0.564 (0.218 ... 1.457)	0.2369
COAD	477	0.640 (0.431 ... 0.949)	0.0265
DLBC	48	1.268 (0.307 ... 5.233)	0.7424
ESCA	163	0.742 (0.454 ... 1.214)	0.2350
GBM	168	0.918 (0.652 ... 1.291)	0.6213
HNSC	503	1.133 (0.867 ... 1.481)	0.3607
KICH	64	3.494 (0.724 ... 16.857)	0.1192
KIRC	541	0.454 (0.331 ... 0.623)	9.83e−07
KIRP	290	0.654 (0.356 ... 1.202)	0.1718
LAML	139	1.514 (0.992 ... 2.311)	0.0542
LGG	530	1.750 (1.226 ... 2.498)	0.0020
LIHC	373	1.627 (1.148 ... 2.307)	0.0062
LUAD	530	1.162 (0.872 ... 1.547)	0.3063
LUSC	496	1.127 (0.860 ... 1.479)	0.3860
MESO	86	0.973 (0.610 ... 1.550)	0.9080
OV	379	1.243 (0.959 ... 1.612)	0.1009
PAAD	179	1.836 (1.207 ... 2.794)	0.0046
PCPG	184	2.777 (0.559 ... 13.791)	0.2116
PRAD	501	1.766 (0.489 ... 6.374)	0.3851
READ	166	0.311 (0.131 ... 0.742)	0.0085
SARC	263	1.243 (0.835 ... 1.849)	0.2838
SKCM	457	1.316 (0.999 ... 1.733)	0.0505
STAD	370	0.890 (0.641 ... 1.235)	0.4842
TGCT	139	2.225 (0.225 ... 22.029)	0.4942
THCA	512	1.557 (0.566 ... 4.288)	0.3914
THYM	119	1.045 (0.274 ... 3.984)	0.9484
UCEC	553	0.864 (0.575 ... 1.298)	0.4807
UCS	57	0.948 (0.487 ... 1.848)	0.8765
UVM	80	1.702 (0.714 ... 4.058)	0.2301

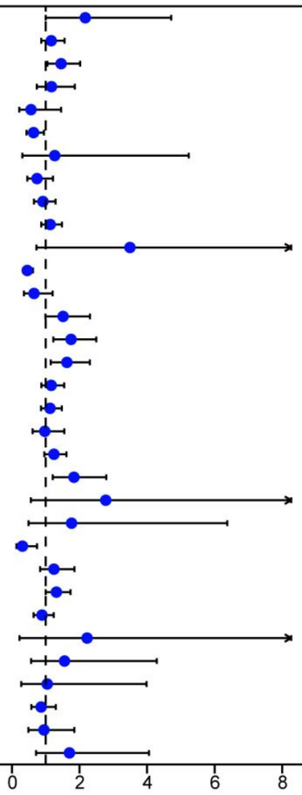

B

DLAT − Progress Free Interval

Group	Total(N)	HR(95% CI)	P value
ACC	79	1.898 (1.011 ... 3.565)	0.0462
BLCA	412	1.321 (0.983 ... 1.775)	0.0649
BRCA	1086	1.289 (0.929 ... 1.789)	0.1290
CESC	306	1.375 (0.862 ... 2.194)	0.1818
CHOL	35	0.685 (0.282 ... 1.666)	0.4041
COAD	477	0.699 (0.492 ... 0.993)	0.0454
DLBC	48	1.039 (0.311 ... 3.471)	0.9506
ESCA	163	0.783 (0.503 ... 1.219)	0.2786
GBM	168	0.824 (0.587 ... 1.158)	0.2649
HNSC	503	1.182 (0.890 ... 1.570)	0.2475
KICH	64	2.779 (0.736 ... 10.487)	0.1315
KIRC	539	0.504 (0.365 ... 0.697)	3.42e−05
KIRP	289	0.838 (0.498 ... 1.411)	0.5069
LGG	530	1.360 (1.033 ... 1.790)	0.0284
LIHC	373	1.443 (1.078 ... 1.931)	0.0138
LUAD	530	1.227 (0.943 ... 1.597)	0.1278
LUSC	497	1.054 (0.763 ... 1.457)	0.7495
MESO	84	1.074 (0.643 ... 1.793)	0.7849
OV	379	1.146 (0.904 ... 1.453)	0.2610
PAAD	179	1.836 (1.236 ... 2.726)	0.0026
PCPG	184	1.883 (0.782 ... 4.536)	0.1581
PRAD	501	1.089 (0.726 ... 1.635)	0.6791
READ	166	0.522 (0.266 ... 1.024)	0.0586
SARC	263	1.089 (0.783 ... 1.515)	0.6112
SKCM	458	1.181 (0.942 ... 1.480)	0.1503
STAD	372	0.862 (0.605 ... 1.226)	0.4081
TGCT	139	1.360 (0.719 ... 2.569)	0.3441
THCA	512	0.769 (0.449 ... 1.315)	0.3374
THYM	119	0.700 (0.293 ... 1.670)	0.4214
UCEC	553	0.976 (0.691 ... 1.379)	0.8912
UCS	57	1.092 (0.572 ... 2.086)	0.7896
UVM	79	1.951 (0.895 ... 4.254)	0.0929

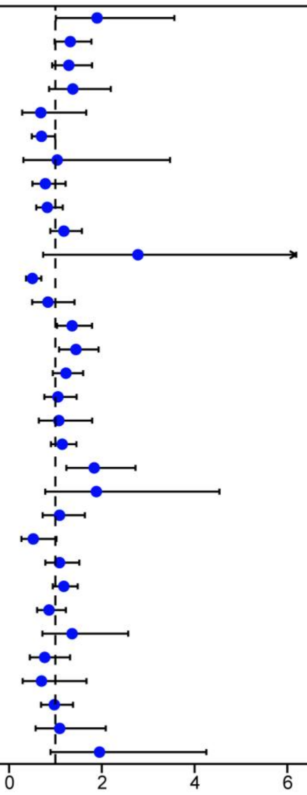

Figure S1 Prognostic Value of DLAT in Pan-Cancer.

(A) The Effect of DLAT on Overall Survival (OS) in Pan-Cancer was analyzed using Cox regression analysis.
(B) The Effect of DLAT on progress free interval (PFI) in Pan-Cancer was analyzed using Cox regression analysis.
